# Effects of nitrogen fertilization and bioenergy crop species on central tendency and spatial heterogeneity of soil glycosidase activities

**DOI:** 10.1038/s41598-020-76837-1

**Published:** 2020-11-12

**Authors:** Min Yuan, Jianjun Duan, Jianwei Li, Siyang Jian, Lahiru Gamage, Kudjo E. Dzantor, Dafeng Hui, Philip A. Fay

**Affiliations:** 1 Department of Agricultural and Environmental Sciences, Tennessee State University, Nashville, TN 37209 USA; 2 Sichuan Provincial Academy of Natural Resource Sciences, Chengdu, 610015 Sichuan China; 3 Guizhou Provincial Key Laboratory for Tobacco Quality, College of Tobacco Science, Guizhou University, Guiyang, 550025 Guizhou China; 4 Department of Biological Sciences, Tennessee State University, Nashville, TN 37209 USA; 5 Grassland Soil and Water Research Laboratory, USDA ARS, Temple, TX 76502 USA

**Keywords:** Microbiology, Biogeochemistry, Environmental sciences

## Abstract

Extracellular glycosidases in soil, produced by microorganisms, act as major agents for decomposing labile soil organic carbon (e.g., cellulose). Soil extracellular glycosidases are significantly affected by nitrogen (N) fertilization but fertilization effects on spatial distributions of soil glycosidases have not been well addressed. Whether the effects of N fertilization vary with bioenergy crop species also remains unclear. Based on a 3-year fertilization experiment in Middle Tennessee, USA, a total of 288 soil samples in topsoil (0–15 cm) were collected from two 15 m^2^ plots under three fertilization treatments in switchgrass (SG: *Panicum virgatum* L.) and gamagrass (GG: *Tripsacum dactyloides* L.) using a spatially explicit design. Four glycosidases, α-glucosidase (*AG*), β-glucosidase (*BG*), β-xylosidase (*BX*), cellobiohydrolase (*CBH*), and their sum associated with C acquisition (*C*_*acq*_) were quantified. The three fertilization treatments were no N input (NN), low N input (LN: 84 kg N ha^−1^ year^−1^ in urea) and high N input (HN: 168 kg N ha^−1^ year^−1^ in urea). The descriptive and geostatistical approaches were used to evaluate their central tendency and spatial heterogeneity. Results showed significant interactive effects of N fertilization and crop type on *BX* such that LN and HN significantly enhanced *BX* by 14% and 44% in SG, respectively. The significant effect of crop type was identified and glycosidase activities were 15–39% higher in GG than those in SG except *AG*. Within-plot variances of glycosidases appeared higher in SG than GG but little differed with N fertilization due to large plot-plot variation. Spatial patterns were generally more evident in LN or HN plots than NN plots for *BG* in SG and *CBH* in GG. This study suggested that N fertilization elevated central tendency and spatial heterogeneity of glycosidase activities in surficial soil horizons and these effects however varied with crop and enzyme types. Future studies need to focus on specific enzyme in certain bioenergy cropland soil when N fertilization effect is evaluated.

## Introduction

Bioenergy crops have the potential to reduce fossil fuel consumption^1^ and the energy crops such as switchgrass (SG: *Panicum virgatum* L.) and gamagrass (GG: *Tripsacum dactyloides* L.) are key for supplying biofuel plant biomass^2,3^. Bioenergy crop yields are enhanced by nitrogen (N) fertilizers^4^, and previous studies frequently focused on aboveground crop yield and less so on belowground features. Nevertheless, N fertilization substantially alters microbial community composition and structure in soil^5^ and consequently impacts soil extracellular enzymes that microbes produced and excreted to the environment^6^. As important proxies to soil health and management^7,8^, soil extracellular enzyme activities mirror soil community’s metabolic requirements and available nutrients^9^. Given the N fertilizer overuse worldwide, investigation of spatial pattern of soil microbial functions such as extracellular enzymes is imperative. Knowledge of spatiotemporal variations of soil extracellular enzymes will further our understanding of soil changes and help develop best management practice under rapid global change.

Extracellular glycosidases are one major soil extracellular enzymes and generally more stable than oxidases in the environment^10–12^. Among extracellular glycosidases, α-glucosidase (*AG*), β-glucosidase (*BG*), β-xylosidase (*BX*), and cellobiohydrolase (*CBH*) are commonly studied to reveal the potential microbial activities associated with fast-turnover organic carbon^13,14^. Thus, these glycosidases have been frequently quantified to study the controls of plant litter decomposition and soil quality. In general, *AG* and *BG* are the most important glycosidases in soils, and their hydrolysis products are source of energy for soil microorganisms. *AG* acts on the α-d-glucoside bonds present in maltose^14^; *BG* catalyzes the hydrolysis of β-d-glucopyranoside and is involved in the saccharification of cellulose^11,15^; *BX* cleaves the β-1,4-linkage of xylan from the non-reducing terminus to release d-xylose and can be used for bioenergy production^16–18^; *CBH* is known to hydrolyze the ends of the cellulose chain and to processively produce glucose or cellobiose as the end product^19^.

In response to N additions, soil extracellular glycosidase activities are altered but the magnitude and direction of the changes vary with enzyme type, soil depth, and crop species. Based on an incubation study of an acidic forest soil, N addition little changed *AG*, *BX,* and *CBH*, but significantly reduced *BG* in the topsoil (2–12 cm)^20^; On the other hand, N fertilization had no effect on these glycosidase activities in topsoil (0–10 cm) in an alpine grassland ecosystem^21^. Whereas, N addition significantly reduced *AG*, *BG*, *BX*, but did not change *CBH* at the deeper soil layers (35–165 cm)^20^. Furthermore, N deposition had minor effects on a wide range of soil extracellular enzyme activities including *BG* and *CBH* in six Chinese forests^22^. Despite no significant N fertilization effect, the significant cropping system effects were identified on *AG* and *BG* such that higher activities were observed in plots under meadow or oat and the lowest under corn and soybean^23^. Similarly, *BG* appeared the highest for sorghum and the lowest for cotton and *BG* was also significantly enhanced by 24% under N fertilization across different cropping systems^24^. Mechanistically, the positive effect of N fertilization on *BG* was likely attributed to plant growth, litter input and associated saprotrophic basidiomycetes^25–27^. N fertilization however also decreased *BG* likely associated with a selective proliferation of soil fungi over bacteria^28^. Despite the variations of N effects and the underlying mechanisms, N fertilization stimulated *AG*, *BG*, *BX* and *CBH* and *C*_*acq*_ based on a meta-analysis^6^.

The spatial distribution (e.g., spatial heterogeneity) of soil extracellular enzymes are evident in the range of centimeters to kilometers^29,30^. The spatial variations of soil extracellular enzymes can be similar across different scales. As an example, the spatial variations of *BG* indexed by coefficient of variation were similar at the microsite (< 100 cm^2^) and plot (> 100 m^2^) scales^29^. Since there were few studies in glycosidases, reports covering hydrolases relevant to other nutrients were discussed here. For instance, different hydrolytic enzymes such as urease, alkaline phosphatase and arylsulfatase involved in N, phosphorus (P) and sulfur (S) acquisitions also showed similar spatial variations^8^. On the other hand, soil invertase, phosphatase, and catalase activities were moderately spatially correlated, whereas urease and dehydrogenase activities were weakly spatially correlated at the county scale^31^. In general, saprotrophic basidiomycetes were regarded to be responsible for the activities and spatial distributions of soil glycosidases such as *BG* and *CBH*^32^, but it was also likely associated with soil physical properties such as plant-stimulated soil pore formation at the 30–150 µm^33^. For instance, *BG* and *BX* activities showed little spatial variations likely related to local abiotic soil properties that are spatially homogeneous^34^. Nevertheless, the spatial heterogeneity of extracellular enzyme activities were more likely evident in grassland and forest soils than agricultural soils^30,35,36^, most likely driven by the contrasting root morphology and chemistry between different plants^37^. To our knowledge, spatial patterns of soil extracellular enzymes in bioenergy crops have not been reported.

The spatial heterogeneity of soil extracellular enzymes under N addition is rarely explored. The only relevant study, to the best of our knowledge, was conducted in a semi-arid Mediterranean shrubland in central Spain^38^. This study showed that high N deposition (50 kg N ha^−1^ year^−1^) tended to homogenize the spatial pattern of soil enzymatic activity including *AG, BG, BX* and *CBH*, and the presence of well-developed soil microbial communities is believed to modulate the effects of high N deposition on soil enzyme activity^38^. However, N fertilization significantly elevated spatial heterogeneity of soil microbial biomass in bioenergy cropland soils^39^, which suggested that, given the generally presumed positive relationship between microbial biomass and soil extracellular enzyme activities^40^, N fertilization may also re-establish spatial heterogeneity of soil extracellular enzymes. In a California grassland, nutrient addition homogenized microbial function (e.g., fungal composition) on infertile soils but increased their spatial variability on fertile soils^41^, suggesting N fertilization effects on spatial heterogeneity of soil microbial biomass and enzyme activities also vary with indigenous site fertility. Noted that despite the positive relationship between microbial biomass and extracellular enzyme activities^40^, such a relationship may not remain valid for spatial patterns of biomass and glycosidases^34^. That means that despite the spatial structures of soil microbial biomass were re-established under N fertilization^39^, the effects of N fertilization on spatial patterns of glycosidases could not be readily derived from the spatial pattern of microbial biomass, and direct observations are needed.

Based on a 3-year long N fertilization experiment located in the campus farm of Tennessee State University, Middle Tennessee, USA, N fertilization plots were selected in SG and GG croplands which were subjected to no-tillage or plowing, and minor mechanical disturbance. Thus, N fertilizer input marked a primary management practice in these bioenergy crop research plots. Based on the fertilization experiment and land use history, this study allowed us to examine how N fertilizations affect central tendency (i.e., plot-level mean) and spatial heterogeneity of four glycosidases (i.e., *AG*, *BG*, *BX*, and *CBH*) and their sum (i.e., *C*_*acq*_) in both SG and GG croplands. We first hypothesized that there was significant N fertilization effect but no significant crop species or interaction of N fertilization and crop species on central tendency of glycosidases, such that N fertilization significantly increased activities of all glycosidases studied, and SG and GG possessed similar activities of glycosidases due to their characteristics of massive root volume^37^; Because N fertilization rebuilt spatial structures of soil microbial biomass and the potentially positive relationship of microbial biomass and extracellular enzymes, our second hypothesis was that in soils that have never been fertilized for years, N fertilization would restructure spatial heterogeneity of *AG*, *BG*, *BX*, *CBH* and *C*_*acq*_. Last, we hypothesized that N fertilization effects on central tendency and spatial heterogeneity varied with enzyme type (e.g. *AG, BG, BX* and *CBH)* due to the unique characteristics of each individual enzyme.

## Materials and methods

### Study site description and experimental design

In 2011, a bioenergy crop field fertilization experiment was established located at the Tennessee State University (TSU) Main Campus Agriculture Research and Education Center (AREC) in Nashville, TN, USA. Prior to the croplands, the land was mowed grassland for several decades with no amendment of fertilizers. The experimental site marks a warm humid temperate climate with an average annual temperature of 15.1 °C, and total annual precipitation of 1200 mm^42^. The crop type and N fertilization treatments were included in a randomized block design^37,39,43,44^. The two crop types were *Alamo* SG (*Panicum virgatum* L.) and GG (*Tripsacum dactyloides* L.). The three N levels included no N fertilizer input (NN), low N fertilizer input (LN: 84 kg N ha^−1^ year^−1^ as urea), and high N fertilizer input (HN: 168 kg N ha^−1^ year^−1^ as urea), and each treatment had four replicated plots with a dimension of 3 m × 6 m. The low N fertilization rate was determined as the optimum N rate to maximize cellulosic ethanol production in established northern latitude grasslands^45^. The high N rate doubled the low rate in order to create appreciable gap and detectable effect between the two levels. The fertilizer was manually applied in June or July each year after cutting the grass. The soil series for the plots is *Armour* silt loam soil (fine-silty, mixed, thermic Ultic *Hapludalfs*) with acidic soil pH (i.e., 5.97) and intermediate organic matter content of 2.4%^39,46^.

### Soil collection and laboratory assay

**Figure 1:**
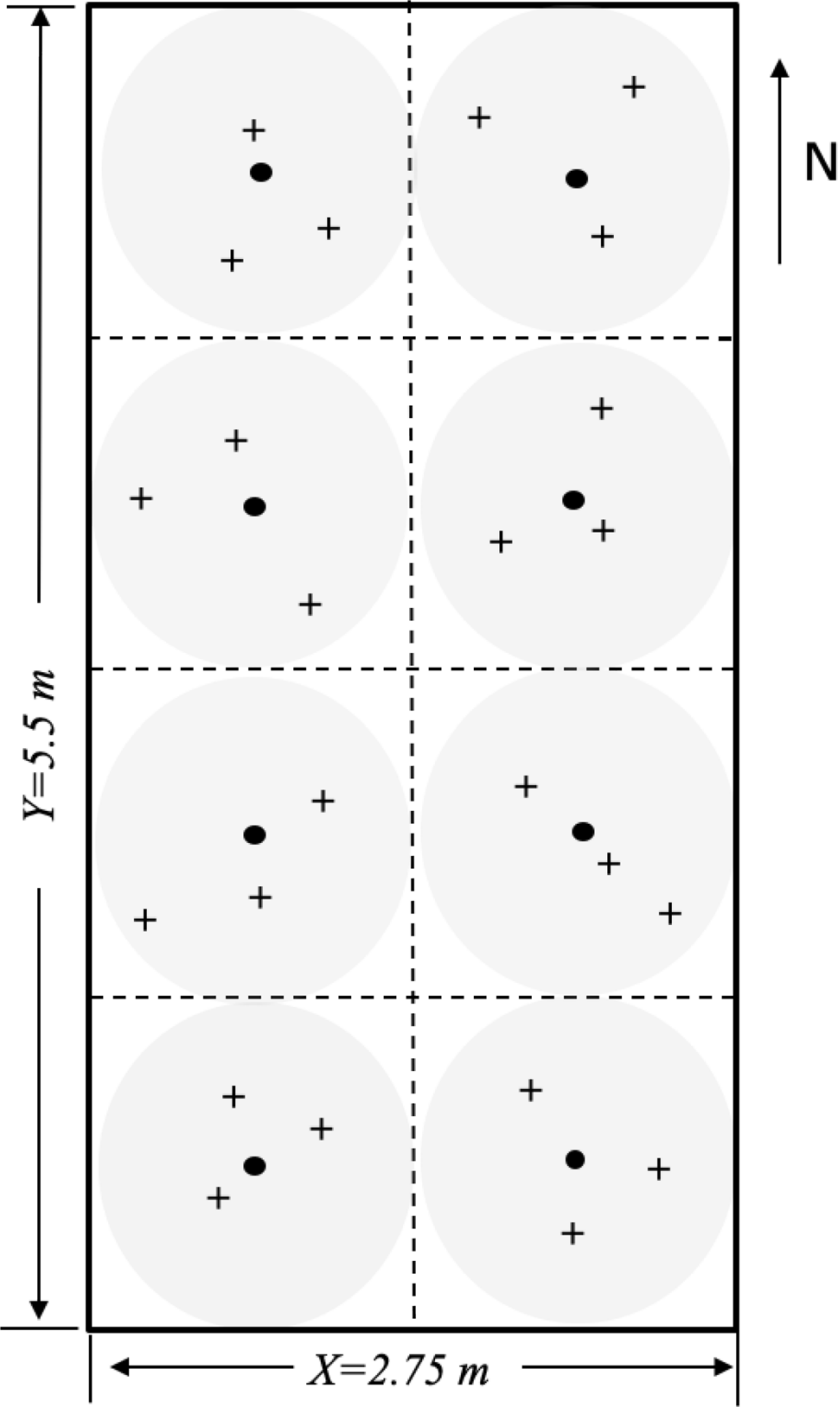
Illustration of an efficient clustered random sampling design within a plot (2.75 m × 5.5 m). The plot was divided into eight subplots (grey zone) and there was a centroid (dark solid circle) in each subplot (1.375 m × 1.375 m), where three soil sampling points (+) were determined from random directions and distances from a centroid in each sampling region (grey area). The extent of an interpolation map was thus determined by the minimum and maximum values at horizontal and vertical axes, and each map can attain its extent less than or equivalent to a plot area.

In this study, soil subsamples were adopted from soil collections based on our former study^39^. Here a brief introduction was presented regarding the former soil collection. On June 6th, 2015, soil samples (0–15 cm) were collected from 12 plots (2 crop × 3 N × 2 replicates). In each plot, soil sampling location was determined in a spatially explicit way accounting for randomization in both sampling direction and distance (Fig. 1). Given this sampling design, the unique x, y coordinates were assigned to each sample. Twenty-four cores were collected from each plot yielding 288 soil cores in 12 plots. Soil samples stored in coolers filled with ice packs were immediately transported to TSU lab and subsequently stored at 4 °C until chemical analysis; and subsamples were stored in − 20 °C for enzymatic assay.

The visible roots and rocks were removed from soil cores by passing through a 2 mm soil sieve prior to chemical analysis and enzymatic assay. For each soil sample, soil gravimetric moisture content was determined by oven drying subsamples for 24 h at 105 °C. Water extractable soil pH was measured given soil: water = 1:5. Four glycosidase activities were quantified by soil fluorimetric enzymatic assay methods in each core. Briefly, soil samples for each plot were assayed for α-glucosidase (*AG*), β-glucosidase (*BG*), β-xylosidase (*BX*), and cellobiohydrolase (*CBH*) using 4-methylumbelliferyl (MUB)-α-d-glucopyranoside, MUB-β-d-glucopyranoside, MUB-β-d-xylopyranoside, and MUB-β-d-cellobioside with concentrations of 200 mmol/L as substrates, respectively, following published protocols^47,48^. Sample suspensions were prepared by placing 1.0 g soil in a 125 ml Nalgene bottle. Acetate buffer (50 mM, pH 5) was added to the bottle and the resulting suspension was homogenized using a Brinkmann Polytron for approximately 1 min. Additional buffer was added to the bottle to bring the final suspension volume to 125 ml.

The plates were placed in an Echotherm incubator at 20 °C, for 18–24 h given enzyme type. The assay of glycosidase activities was conducted on black 96-well microtiter plates. The assay design included reference standards (eight wells) and quench controls (eight wells per sample) added to each plate. The 10 µM MUB was used as the reference standard for *AG, BG, BX* and *CBH*. Quench control wells contained 200 µl of sample suspension and 50 µl of the reference standard. The assay was incubated at 20 °C. The reactions were terminated by adding 10 µl of 1.0 M NaOH to each well. Fluorescence was measured using a Molecular Devices (Multi-Mode Microplate Reader, FilterMaxF5) with excitation set to 365 nm and emission set to 460 nm. All enzyme activities were calculated as µmol activity h^−1^ g soil^−1^. A total of 1152 enzymatic activity data were collected for 288 soil samples and 4 enzymes. Laboratory tests were conducted and specific protocols were optimized to secure sufficient soil mixing. As a result, the variation of each measurement (i.e., coefficient of variation) in multiple tests ranged from 2 to 8% based on our protocol.

### Statistical analysis

We use both descriptive and geospatial analytical methods to illustrate the central tendency and spatial heterogeneity of enzymes assayed. Mean, frequency distribution, plot-level variance and with-plot coefficient of variation (CV) were estimated to describe central tendencies and variations for enzyme activities in each plot. The two-way ANOVA was used to test whether N fertilization, crop species and their interaction significantly affected each enzyme. To avoid the pseudo-replication impacts, the plot means were used in the two-way ANOVA test. The statistically significant level was set at P < 0.05.

Cochran's C test was performed to test the assumption of variance homogeneity. The test statistic is a ratio that relates the largest empirical variance of a particular treatment to the sum of the variances of the remaining treatments. The theoretical distribution with the corresponding critical values can be specified. Soil properties that exhibited non-normal distributions were log-transformed to better conform to the normality assumption of the Cochran’s C test^49,50^.

The sample size required in a research plot can be determined quantitatively under given desired sampling error^51^. That is, under a desired sampling error, the sample sizes derived can be used to evaluate the plot-level variations between different research plots. In this study, the sample size requirement ($N$) in each plot was derived given specified relative error ($\gamma$), which was defined as the ratio of error term (${t}_{0.975}\times \frac{s}{\sqrt{n}}$) over plot mean ($\overline{\text{X}}$) with a range of 0–100% (Eqs. 1–3). To evaluate how sample size requirement varied with N fertilization or crop types at certain relative error, the average of sample size ($N$) in two plots was derived and plotted. Under a relative error of 10%, the sample sizes were also derived from each plot and compared between different plots. For comparison, the higher sample size the greater plot-level variation under the same relative error.

1 $$\text{CI}=\overline{\text{X}}\pm {t}_{0.975}\times \frac{s}{\sqrt{n}}$$

2 $$\upgamma =\frac{{t}_{0.975}\times \frac{s}{\sqrt{n}}}{\overline{\text{X}}}= {t}_{0.975}\times \frac{CV}{\sqrt{N}}$$

3 $$\text{ln}(\text{N})=2\times \text{ln}( {\text{t}}_{0.975} \times \text{CV})-2\times \left(\upgamma \right) $$

where $$\text{CI}, \overline{\text{X}},\text{ s},\text{ n},\text{ N},\text{ CV},\text{ and} \; \upgamma $$ denote confidence interval, plot means, plot standard deviation, sample number (n = 24), coefficient of variation, sample size requirement and relative error, respectively. $${\text{t}}_{0.975}$$= 1.96. The log-transformed sample size requirement ($$N)$$ has a negative linear relationship (i.e. slope = 2) with the log-transformed relative error $$\left(\gamma \right)$$.

### Geostatistical analysis

Three different geostatistical tools were applied to describe the spatial structure of soil exoenzyme activities within and among plots. The methods were briefly described below and more details could be found in Li^52^. First, trend surface analysis (TSA) is the most common regionalized model in which all sample points fit a model that accounts for the linear and non-linear variation of an attribute. Relationships between soil properties and x and y coordinates of their measurement location within the sampling plots are estimated with the trend surface model (Eq. 4):

4 $$\text{Soil}\; \text{property}\; \text{value }= {\beta }_{0}+{\beta }_{1}x+{\beta }_{2}y+{\beta }_{3}xy+{\beta }_{4}{x}^{2}+{\beta }_{5}{y}^{2}$$

The presence of a trend in the data was determined by the significance of any of the parameters ${\beta }_{1}$ to ${\beta }_{5}$ , while the ${\beta }_{0}$ was the intercept^53,54^ . Linear gradients in x or y directions were indicated by the significance of ${\beta }_{1}$ or ${\beta }_{2}$. A significant ${\beta }_{3}$ indicated a significant diagonal trend across a plot. Significant ${\beta }_{4}$ and ${\beta }_{5}$ parameters indicated a more complex, nonlinear spatial structure such as substantial humps or depressions. Trend surface regressions were estimated using *R* program^55^. Model parameters were determined to be significant at a level of P < 0.05.

Second, residuals from the trend surface regressions were saved for subsequent spatial analysis using a Moran’s I index^56^. The Moran's I analysis^57–59^ was used to quantify the degree of spatial autocorrelation that were present in each plot. The resulting local Moran's I statistics is in the range from – 1 to 1 With a positive Moran's I values indicating similar values (either high or low) are spatially clustered, and a negative Moran's I values indicating neighboring values are dissimilar. No spatial autocorrelation or spatial randomness was reached with a Moran's I value of 0. Given that the observed Moran’s I value is beyond the projected 95% confidence interval at a certain distance, this is identified as a significant autocorrelation. In this study, correlograms were produced for soil variables in all plots given a range of 0–5.5 m with 0.25 m incremental interval.

Third, an ordinary kriging method was usually used to produce maps which offered direct and visual assessments from which to compare the spatial distributions of the soil properties among the plots^60^. The ordinary kriging method required a large sample size (i.e., a few hundred or more) in order to achieve reliable interpretation maps^60^. Due to the fine-scale sampling region (1.375 × 1.375 m) and a relatively small sample size per plot (n = 24), inverse distance weighting (IDW) interpolation was used in this study. The IDW maps were formerly used to distinguish the effects of different land uses on spatial distributions of soil biogeochemical features in South Carolina, USA^52^. Briefly, the weights for each observation were inversely proportional to the power of its distance from the location being estimated. Exponents between 1 and 3 we typically used for IDW. Tests with different IDW exponents indicated that 2 was optimal with data collected in this study, as the exponent of 2.0 showed the best fit between estimated values and actual data in cross-validation tests^61^. ArcGIS 10.6 (ESRI, USA) was used to generate the IDW maps and perform cross-validations.

## Results

### Central tendencies and within-plot variances

There were significant main and interactive effects of N fertilization and crop species on *BX* activity (Table 1), and post hoc tests showed that relative to unfertilized treatment (NN), N fertilization treatments significantly escalated *BX* activity by 14% (LN) and 44% (HN) in SG (Table 2). There were also significant effects of crop species on the activities of *BG* and *BX* (Table 1) and the effect of crop species on *CBH* was marginally significant (P = 0.056; Table 1). Relative to SG, GG was higher by 15%, 31%, 32%, and 39% in *BX, BG, C*_*acq*_ and *CBH,* respectively (Table 2). There were no significant effects of N fertilization or interaction of N fertilization and crop species on activities of *AG, BG, CBH* or *C*_*acq*_ (Table 1).

**Table 1:** P-values of two-way ANOVA tests for the main and interactive effects of N fertilization and crop species on *AG, BG, BX, CBH*, and *C*_*acq*_ (µmol g^−1^soil h^−1^).

Enzyme type	Fertilization	Crop	Fertilization * crop
*AG*	0.1014	0.7691	0.0565
*BG*	0.222	**0.0099**	0.3961
*BX*	**0.0077**	**0.0233**	**0.0337**
*CBH*	0.5672	0.0558	0.682
*C*_*acq*_	0.3014	0.0178	0.4603

Bold numbers denote significant treatment effects at *P* < 0.05.

*AG*: α-glucosidase; *BG*: β-glucosidase; *BX*: β-xylosidase; *CBH*: cellobiohydrolase; *C*_*acq*_: the sum of *AG, BG, BX* and *CBH.*

**Table 2:** Means (± SE) of *AG, BG, BX, CBH,* and *C*_*acq*_ (µmol g^−1^soil h^−1^) under three N fertilization treatments (NN, LN and HN) in two bioenergy croplands (SG and GG).

Crop	Fertilization	*AG*	*BG*	*BX*	*CBH*	*C*_*acq*_
SG	NN	1.34 ± 0.22^a^	75.56 ± 01.91^a^	5.58 ± 0.20 ^b^	24.15 ± 01.15^a^	106.62 ± 00.79^a^
	LN	1.24 ± 0.05^a^	94.83 ± 12.27^a^	6.35 ± 0.37^ab^	30.72 ± 05.16^a^	133.14 ± 17.75^a^
	HN	1.90 ± 0.01^a^	108.59 ± 07.95^a^	8.03 ± 0.23^a^	36.50 ± 03.05^a^	155.01 ± 10.76^a^
GG	NN	1.58 ± 0.19^a^	119.64 ± 24.18^a^	8.13 ± 0.11^a^	41.78 ± 14.81^a^	171.13 ± 39.30^a^
	LN	1.47 ± 0.09^a^	122.62 ± 12.47^a^	6.33 ± 0.09^ab^	41.96 ± 09.55^a^	172.38 ± 22.02^a^
	HN	1.46 ± 0.25^a^	124.45 ± 12.49^a^	8.40 ± 1.26^a^	43.11 ± 10.09^a^	177.42 ± 24.09^a^

SG: switchgrass; GG: gammagrass; NN: No nitrogen fertilizer input; LN: low nitrogen (84 kg N ha^−1^ year^−1^ in urea); HN: High nitrogen (168 kg N ha^−1^ year^−1^ in urea). In each column, different lowercase letters denote significant difference between fertilization treatments at P < 0.05 (N = 2).

**Figure 2:**
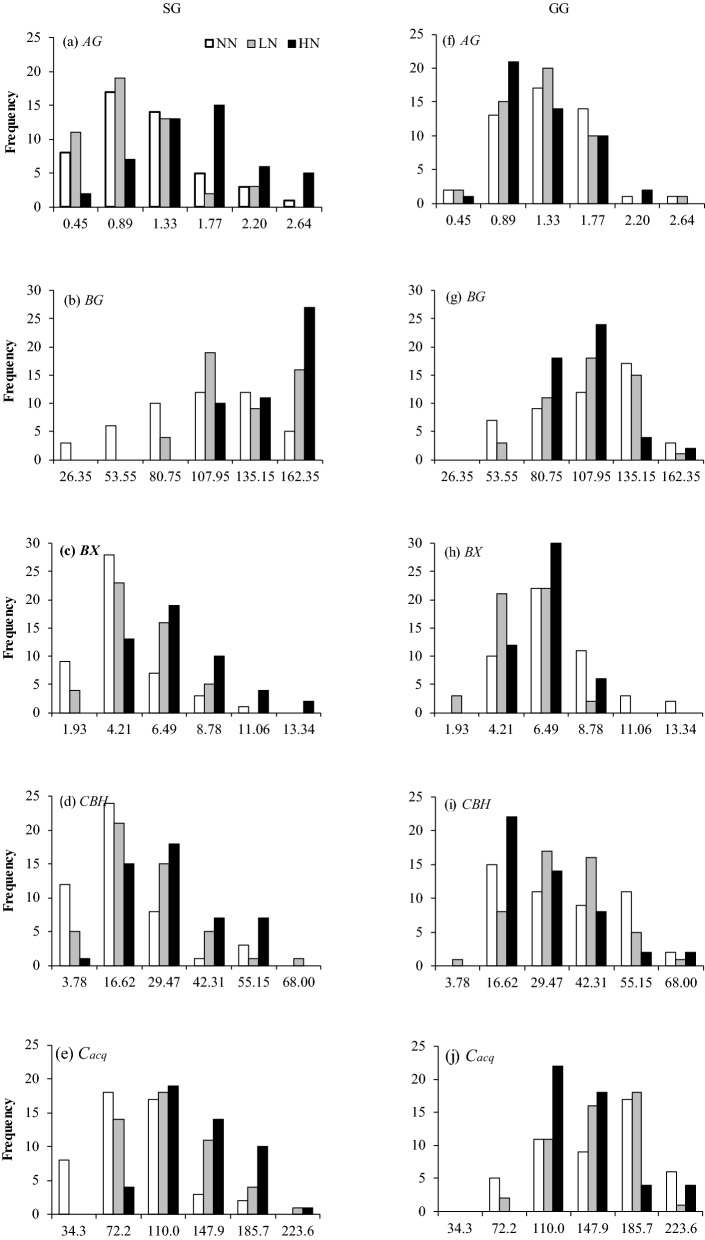
Frequency histograms of *AG, BG, BX, CBH* and *C*_*acq*_ under three N fertilization treatments (NN, LN and HN) in two bioenergy croplands (SG and GG). The number on the x-axis (i.e. 0.45, 0.89 in (**a**) represents a range of (0.00, 0.45) and (0.45, 0.89), respectively. The abbreviations are referred to Tables 1 and 2.

The frequency diagrams of four glycosidase activities and *C*_*acq*_ showed nearly normal distributions under all treatments in two croplands except *BG* under HN in SG (Fig. 2). The frequency distributions contrasted substantially among different N fertilization treatments for SG, whereas, the frequency distributions of different N fertilization treatments showed relatively similar ranges for GG (Fig. 2). Remarkably, for *BX* in SG, NN appeared to have the highest frequency in lower values, whereas, HN had the highest frequency in higher values. This frequency distributions between NN and HN was consistent with a significantly escalated *BX* activity in HN compared to NN (Table 2). On the other hand, the Cochran’s C tests showed that N fertilization little changed plot-level variation for most glycosidase activities in both bioenergy croplands, except that HN induced the highest plot-level variance for *AG* in SG (Table 3).

**Table 3:** Comparison of the variances and Cochran’s C test results for *AG, BG, BX, CBH,* and *C*_*acq*_ (µmol g^−1^soil h^−1^) under three N fertilization treatments (NN, LN and HN) in two bioenergy croplands (SG and GG).

Crop	Fertilization	Plot	*AG*	*BG*	*BX*	*CBH*	*C*_*acq*_
SG	NN	P1	0.37	698.1	3.93	242.7	1459.8
		P2	0.15	450.1	3.33	119.9	987.6
	LN	P1	0.26	539.0	3.18	98.3	1081.9
		P2	0.13	739.9	2.56	217.8	1789.8
	HN	P1	0.50	338.0	5.46	210.7	1086.6
		P2	0.24	704.2	6.51	163.7	1530.1
Cochran's test		*C* value	0.30	0.21	0.26	0.23	0.26
		*p*-value	0.03	0.90	0.16	0.51	0.60
GG	NN	P1	0.15	815.5	4.09	109.1	1632.9
		P2	0.17	421.9	5.05	169.6	1006.0
	LN	P1	0.12	750.0	3.97	136.1	1267.2
		P2	0.16	371.4	1.03	130.4	788.1
	HN	P1	0.07	398.7	1.59	191.8	1119.5
		P2	0.17	792.5	3.37	186.9	1710.1
Cochran's test		*C* value	0.20	0.20	0.23	0.26	0.21
		*p*-value	1.00	0.52	0.14	1.00	0.57
Total Cochran's test		*C* value	0.20	0.20	0.12	0.15	0.33
		*p*-value	0.00	0.00	1.00	0.11	1.00

The abbreviations are referred to Tables 1 and 2.

**Figure 3:**
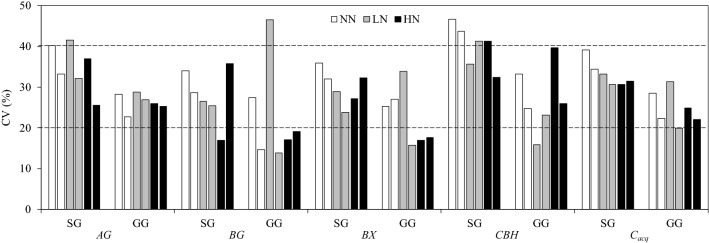
Within-plot CVs of *AG, BG, BX, CBH* and *C*_*acq*_ under three N fertilization treatments (NN, LN and HN) in two bioenergy croplands (SG and GG). The dashed lines represent a CV of 20% and 40%. Different lowercase letters denote significant difference in CV between fertilization treatments and different uppercase letters between crop species for each enzyme at P < 0.05. The abbreviations are referred to Tables 1 and 2.

The within-plot CVs of four glycosidases and *C*_*acq*_ ranged from 15 to 47% in all treatments (Fig. 3). The CVs of *AG*, *CBH* and *C*_*acq*_ were higher in SG than those in GG (Fig. 3). In 12 plots, the number of plots with CVs larger than 40% for *AG*, *BG, BX, CHB* and *C*_*acq*_, were 2, 0, 0, 4 and 2 in SG, and 0, 1, 0, 1 and 0 in GG, respectively; Accordingly, the number of plots with CVs less than 20%, were 0, 1, 0, 0 and 0 in SG, and 0, 4, 3, 1 and 0 in GG, respectively.

**Figure 4:**
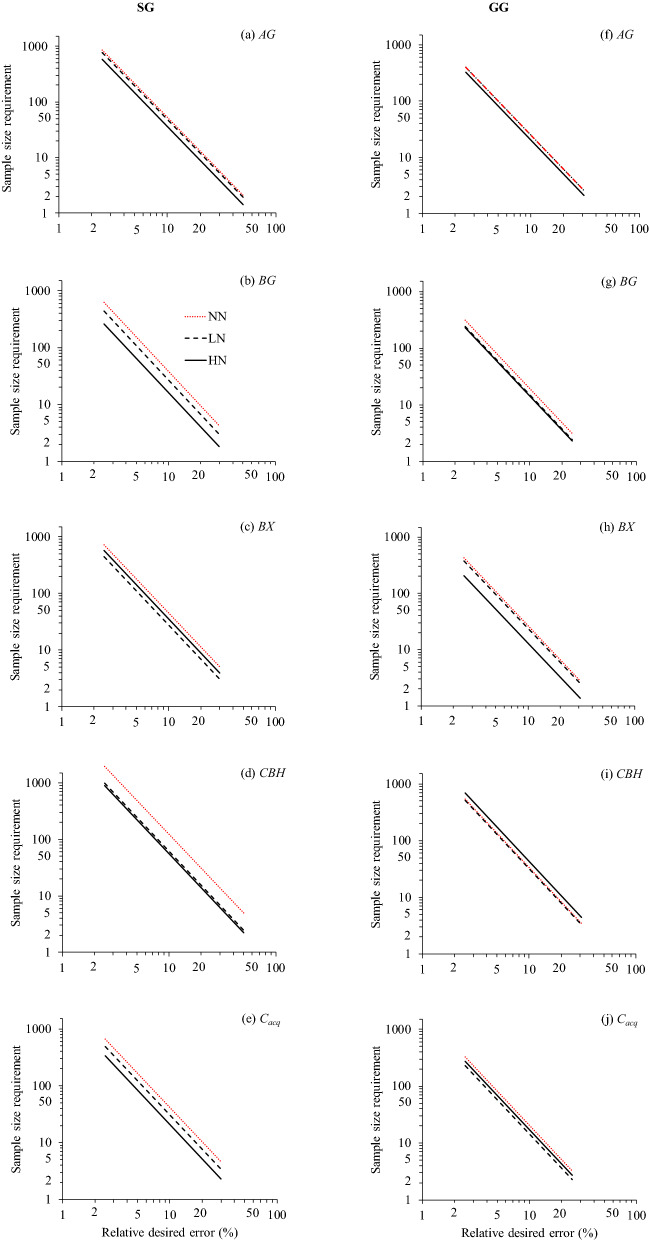
Plots of log transformed sample size requirements (SSR) and desired relative errors *AG, BG, BX, CBH* and *C*_*acq*_ under three N fertilization treatments (NN, LN and HN) in two bioenergy croplands (SG and GG). NN: red dotted line; LN: black dotted line; and HN: black solid line. The log scale was applied on both axes. The abbreviations are referred to Tables 1 and 2. SSR denotes the average of two plots in each treatment.

The sample size requirement (SSR) for all enzymes was generally higher for NN than LN or HN in both croplands, except *CBH* in GG (Fig. 4). The plotted lines of SSR against relative sampling error departed from one to another in SG in a much wider extent than those in GG for all glycosidases except *BX* (Fig. 4). In general, a larger number of samples were required in SG than that in GG for all glycosidases under the same desired relative error. Given the same desired sampling error of 10%, the sample size required were always larger in SG than that in GG; A total of 123 samples were required for *CBH* under NN in SG and only 14 samples were required for *BG* under HN in GG to achieve the desired error of 10% in both plots (Table 4).

**Table 4:** Sample size requirement for *AG, BG, BX, CBH,* and *C*_*acq*_ (µmol g^−1^soil h^−1^) under the relative error of 10% under three N fertilization treatments (NN, LN and HN) in two bioenergy croplands (SG and GG).

Enzyme	Crop type	Relative error, %	NN	LN	HN
*AG*	SG	10	53	49	36
*AG*	GG	10	25	25	20
*BG*	SG	10	38	27	16
*BG*	GG	10	19	15	14
*BX*	SG	10	45	28	36
*BX*	GG	10	26	24	13
*CBH*	SG	10	123	61	55
*CBH*	GG	10	33	32	43
*C*_*acq*_	SG	10	41	30	21
*C*_*acq*_	GG	10	20	14	17

Each sample size denotes the average of sample size in two plots under the same treatment. The abbreviations are referred to Tables 1 and 2.

### Surface trend, autocorrelation and spatial map

Trend surface analysis results showed only a few significant linear or nonlinear trends in each plot, and about half of plots showed no significant linear or nonlinear trends (Table 5; Table S1). Given the detected significant surface trends, there was a contrasting pattern of surface trend with N fertilization between SG and GG (Table 5). In SG, there were no significant linear or nonlinear trends in any of HN plots for all enzymes. Relative to NN plots, there were more significant linear or nonlinear surface trends of *AG* and *BG* in LN plots, whereas there were comparable number of surface trends of *BX*, *CBH*, and *C*_*acq*_ between NN and LN plots (Table 5). In GG, there were no significant linear or nonlinear trends in any of NN plots for all enzymes except *AG*, and there were no any significant surface trends in any plot for *BX* or in any of NN and LN plots for *CBH*. Relative to NN plots, there were more significant linear or nonlinear surface trends of *BG*, *CBH*, and *C*_*acq*_ in LN or HN plots; whereas there were comparable or larger number of surface trends of *AG* in NN than those in LN or HN plots (Table 5). Under the same treatment, the number of significant linear or nonlinear trends varied between the two replicated plots, and in all cases except *AG* in LN plot in GG, there was significant surface trends in one plot, but none in another plot (Table S1).

**Table 5:** The number of significant regression coefficients of trend-surface analysis for *AG, BG, BX, CBH,* and *C*_*acq*_ (µmol g^−1^soil h^−1^) under three N fertilization treatments (NN, LN and HN) in two bioenergy croplands (SG and GG).

Crop type	Enzyme	NN	LN	HN
SG	*AG*	0	2	0
	*BG*	0	1	0
	*BX*	2	2	0
	*CBH*	3	3	0
	*C*_*acq*_	2	2	0
GG	*AG*	2	1	2
	*BG*	0	1	2
	*BX*	0	0	0
	*CBH*	0	0	2
	*C*_*acq*_	0	1	2

Values represent the sum of significant regression coefficients in two replicated plots under each treatment. The regression coefficients denote parameters ${\beta }_{1}$ to ${\beta }_{5}$ in Eq. (4). The significant coefficients of trend-surface analysis for each plot was presented in Table S1. The abbreviations are referred to Tables 1 and 2.

**Figure 5:**
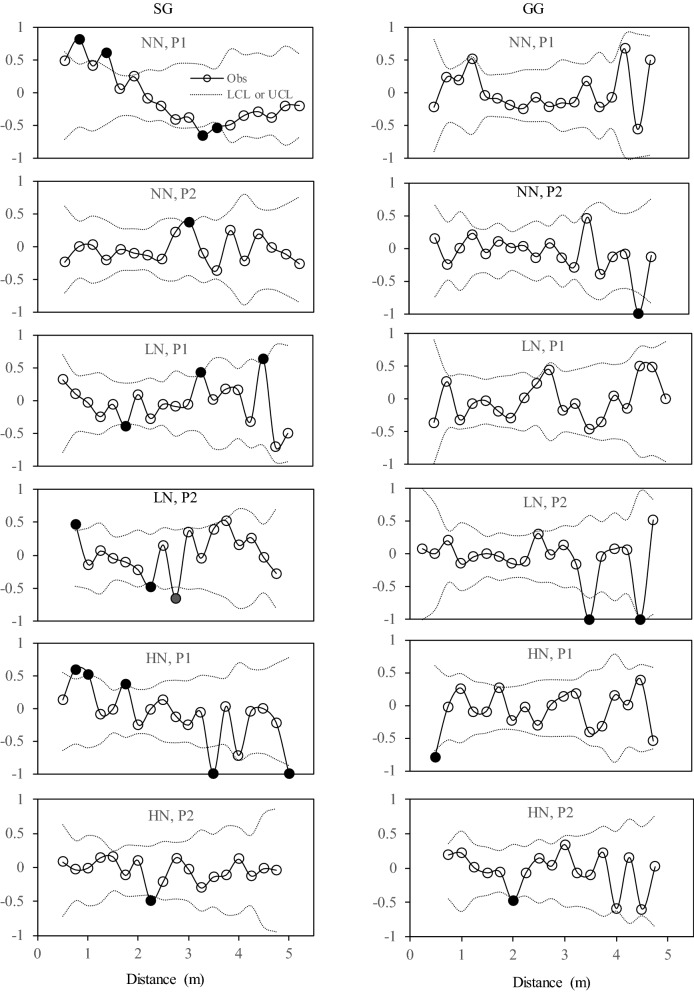
Correlograms of Moran’s I for *AG* under three N fertilization treatments (NN, LN and HN) in two bioenergy croplands (SG and GG). Filled circles, positioned beyond the upper and lower dashed lines, represent positive or negative Moran’s I values that exhibited significant autocorrelation. Obs: observations; LCL: low confident limit; and UCL: upper confident limit. Abbreviations are referred to Tables 1 and 2.

The number and distance of significant spatial autocorrelations varied with N fertilization treatments, bioenergy croplands, and among variables (Table 6). The number of significant spatial autocorrelations in SG was identified more frequently than that in GG for *AG*, *BX*, *CBH*, and *C*_*acq*_ across three N fertilization treatments. Compared to NN, fertilized treatments (LN and HN) possessed a higher number of significant spatial autocorrelations for *BG*, *BX*, *CBH* in SG, and for *AG*, *BX*, *CBH* in GG. The distance of significant spatial autocorrelation appeared to be positive or negative in any plot for any enzyme studied. The distances in which the significant spatial autocorrelations appeared ranged from − 5.25 to 5 m across all enzymes. Relative to other enzymes, *AG* showed significant spatial autocorrelations in more diverse distances in almost all plots except P1 in NN and LN plots in GG (Fig. 5).

**Table 6:** Summary of significant distance for spatial dependence based on Moran’s I values for *AG, BG, BX, CBH,* and *C*_*acq*_ (µmol g^−1^soil h^−1^) under three N fertilization treatments (NN, LN and HN) in two bioenergy croplands (SG and GG).

Crop	Fertilization	Plot	*AG*	*BG*	*BX*	*CBH*	*C*_*acq*_
SG	NN	P1	0.75, 1.25, − 3.00, − 3.25		0.75, 5.00		0.75, 5.00
		P2	2.75	2.75	− 2	− 4.75	
	LN	P1	− 1.75, 3.25, 4.50	− 1.75		− 3.25	
		P2	0.75, − 2.25, − 2.75	0.75, − 2.25	0.75, − 4.50, − 5	0.75, − 2.25, − 2.75	0.75; − 2.25
	HN	P1	0.75, 1.00, 1.75, − 3.50, − 5.00	− 3.50, − 4.00	0.75, − 3.50, 4.00, − 5.00	0.75, 1.00, 3.25, − 3.50	0.75, 1.00, 3.25, − 3.50
		P2	− 2.25				
GG	NN	P1					
		P2	− 4.5	3.50, − 5.00			3.50, − 5.00
	LN	P1		0.50, − 4.00, − 4.50, − 4.75, − 5.25	3.75, − 4.50		0.5
		P2	− 3.5		− 0.75	− 3.75,4.25	
	HN	P1	− 0.50, 1.00		3.25	− 2	
		P2	− 2.00, − 4.00	0.5			0.5

The unit of the distance for spatial dependence is meter. The abbreviations are referred to Tables 1 and 2.

**Figure 6:**
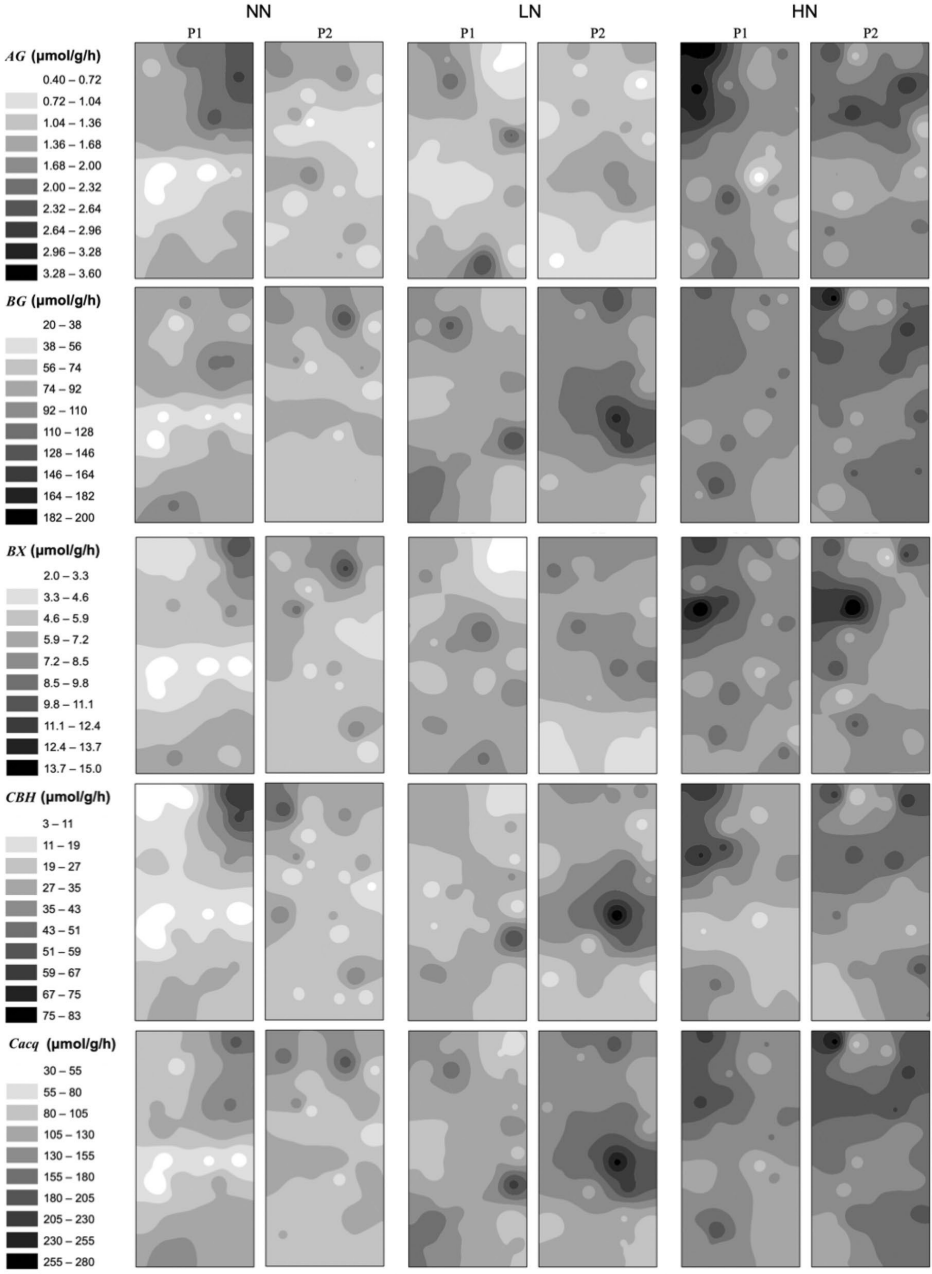
Spatial distributions of *AG*, *BG, BX, CBH* and *C*_*acq*_ activity in soils under three N fertilization treatments (i.e. NN, LN and HN) in SG. The interpolation maps were produced by inverse distance weighting (IDW) method using ArcGIS software by Esri (version 10.2.1, http://www.esri.com). The abbreviations are referred to Tables 1 and 2.

**Figure 7:**
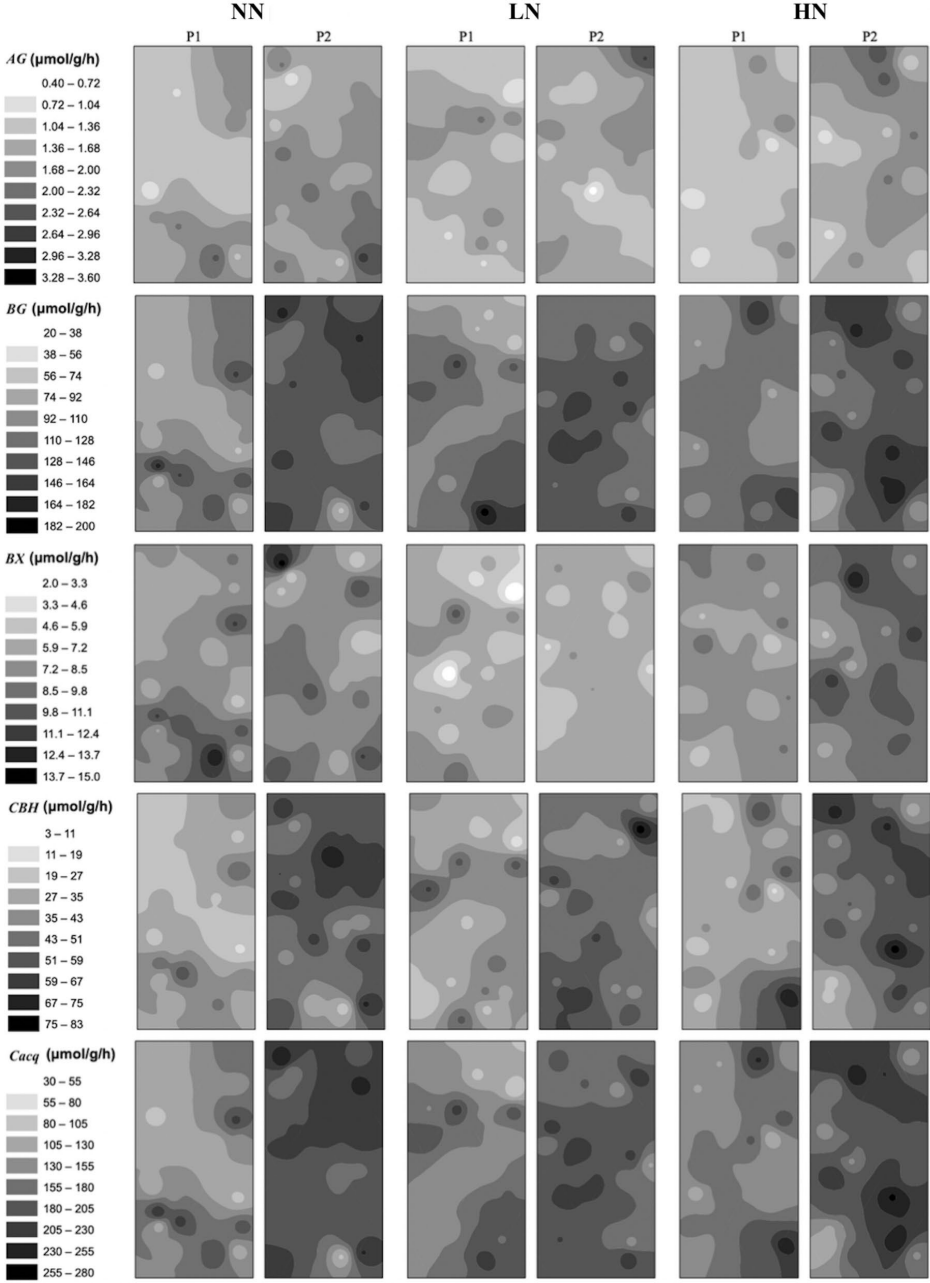
Spatial distributions of *AG*, *BG, BX, CBH* and *C*_*acq*_ activity in soils under three N fertilization treatments (i.e. NN, LN and HN) in GG. The interpolation maps were produced by inverse distance weighting (IDW) method using ArcGIS software by Esri (version 10.2.1, http://www.esri.com). The abbreviations are referred to Tables 1 and 2.

With the same scale for each enzyme in two crops, the IDW maps of all enzymes exhibited higher activities (e.g., darker color) in GG than those in SG, and this was true in unfertilized and fertilized plots (i.e., NN, LN and HH) (Figs. 6 and 7). In SG, all IDW maps exhibited low to high activities (e.g., shallower and gradually darker colors) from NN plots, through LN, to HN plots (Fig. 6). Also, the contrast between dark and shallow color regimes in a plot were more pronounced in LN or HN relative to NN, and this was particularly evident for *BX* (Fig. 6). In GG, the IDW maps exhibited different patterns from those in SG. Large variations of color regime were identified among different enzymes with darker color for *BG*, *CBH* and *C*_*acq*_ (Fig. 7). The color regimes were comparable and evident among all plots for each enzyme (Fig. 7).

## Discussion

### N fertilization elevated BX activity in switchgrass cropland soils

Our current study identified that only *BX* activity was significantly affected by N fertilization. This only partially supported our first hypothesis that all studied glycosidases would increase with N fertilization. The responsiveness of *BX* may lie in several possible mechanisms. First, N fertilization could increase production and excretion of *BX* given the elevated relative abundance of Gram-negative bacteria in cropland soils^62,63^ because of the close association of *BX* with Gram-negative bacteria^64^. Second, *BX* was found out to be significantly correlated with soil C contents but not significantly correlated with microbial biomass^65^. In our plots, N fertilization significantly increased SOC by up to 16% but little changed microbial biomass^39^. The positive response of *BX* thus was likely associated with the elevated SOC stock under N fertilization. Third, N fertilization stimulated plant growth and subsequent stimulation of microbial activity in our plots^37^, this likely contributed to the elevated *BX* but it remained unclear why other glycosidases studied showed no positive response to N fertilization. One possible explanation for the lack of response in *CBH* may lie in the N fertilization effect on the Gram-positive bacteria, fungi and the actinomycetes^63,66,67^, which correlated with *CBH*^68^. Interestingly, the detectable response of *BX* suggested that the fundamental attributes of enzymatic reactions could be extrapolated from molecule through community to ecosystem scales^69^.

In addition, *BX* and *BG* were significantly and *CBH* was marginally significantly higher (by > 30%) in GG than that in SG. This rejected part of our first hypothesis that there was no significant difference of glycosidase activities between SG and GG. Despite similar characteristics of both SG and GG roots, i.e., massive root volume, the contrasting root chemistry of the two plants may induce different strategy to compete with soil microbes for nutrients^70^. Given the more structurally complex nature of GG root than SG root^37^, this may slow GG root to acquire readily available N (e.g., ammonium or nitrate) and thus result in high nutrient availability to microbes. The strategy of microbial nutrient acquisition may thus shift from the control by nutrient deficiency to nutrient abundance, resulting in less *BX* production and expression under N fertilization in GG. Besides the crop species, the effect of N fertilization on glycosidases were also reported to co-vary with other factors, such as soil depth and sampling location (rhizosphere vs. bulk soil)^71,72^ and soil and ecosystem types^6^.

Based on the post hoc tests of the interaction term, *BX* activity was significantly escalated under N fertilization in SG. That is, the positive response of *BX* to N fertilizer appeared much less in GG than SG. This may lie in several different mechanisms. First, relative to SG, the percentage increases of SOC under N fertilization were much smaller in GG^44^, which could limit the response of *BX* given the relationship of *BX* and soil C contents^65^. Second, soil microbial biomass showed no significant change in SG but significantly increased under N fertilization in GG^39^. We speculate that despite the increased microbial biomass, the substrate availability to microbes may have been limited due to no change of SOC under N fertilization^44^, which could result in microbial C limitation and subsequently a constraint on enzyme productions.

### N fertilization restructured spatial heterogeneity of glycosidases

In support of our second hypothesis, N fertilization resulted in more pronounced spatial heterogeneity of glycosidases in both bioenergy croplands. Despite tremendous efforts committed to avoid nutrient hotspots due to uneven fertilizer application, the elevated spatial heterogeneity were still likely induced by fertilizer application in bioenergy crops because manual spread of N fertilizers would create significant irregularity of nutrient deposit and clusters^39,44^, and consequently favor the formation of hotspots of soil microbial communities^73^. In these hotspots, microbes grew faster and SOC appeared high so that these conditions may eventually create hotspots of microbial functions, such as greater extracellular enzyme activities. Consistent with this speculation, the positive effect of N fertilization on spatial heterogeneity of SOC were also found in the same field experiment^44^. This intricate associations of bulk soil C and glycosidases were also supported by their significant correlation coefficients (P < 0.05; Table 7). Meanwhile, there was no significant correlations between microbial biomass C and any of these glycosidases studied (Table 7). The correlation between SOC and glycosidase and no correlation between MBC and glycosidase are consistent with the findings revealed in Waldrop, Balser^65^. These suggested that the N fertilization induced heterogeneity of glycosidases may be largely moderated by the microbial substrate availability over space (e.g., SOC), not necessarily by the microbial abundance (e.g., MBC). This strategy was consistent with the recent discovery of the negative correlation between maximum microbial growth rate and soil extracellular hydrolytic enzymes under high resource conditions^74^. With readily available nutrients (e.g., nitrate and ammonium) under N fertilization, the resource acquisition strategists invest heavily in extracellular enzyme production while other microbial groups (e.g., growth strategist and maintenance strategist) either compete for fast growth or limit investment in both enzymes and growth^74^. Noted that a positive effect of N fertilization on spatial heterogeneity of MBC was also found in the same experiment^39^, suggesting a decoupling of spatial distributions of MBC and glycosidases under N fertilizations. Collectively, these results suggested that the change and distribution of glycosidases may primarily be driven by the substrate availability for microbes, not the abundance of microbes themselves.

**Table 7:** Pearson correlation coefficients between SOC, TN, C/N, MBC, MBN, MBC/MBN, and glycosidase activities (i.e., *AG, BG, BX, CBH*) under three N fertilization treatments (NN, LN and HN) in two bioenergy croplands (SG and GG).

	SOC	TN	C/N	MBC	MBN	MBC/MBN	*AG*	*BG*	*BX*	*CBH*
SOC	1.00									
TN	**0.92**	1.00								
C/N	**0.28**	− 0.12	1.00							
MBC	**0.19**	**0.21**	− 0.01	1.00						
MBN	**0.18**	**0.21**	− 0.04	**0.36**	1.00					
MBC/MBN	− 0.03	− 0.04	0.00	**0.41**	− 0.64	1.00				
*AG*	− 0.05	− 0.09	0.11	− 0.04	− 0.08	0.03	1.00			
*BG*	**0.19**	**0.23**	− 0.07	0.05	**0.13**	− 0.11	**0.47**	1.00		
*BX*	0.09	0.07	0.07	0.01	0.10	− 0.10	**0.54**	**0.54**	1.00	
*CBH*	**0.21**	**0.27**	− 0.12	− 0.02	0.08	− 0.10	**0.53**	**0.69**	**0.53**	1.00

Bold values denote significant correlation coefficients at P < 0.05.

### N fertilization effects on central tendency and spatial heterogeneity of glycosidases varied with enzyme type

In support of our third hypothesis, this study showed that the N fertilization effects on central tendency and spatial heterogeneity of glycosidases varied with enzyme type. This result is not surprising due to the generally high indigenous soil heterogeneity. N fertilizer applied in each point in a plot impact glycosidase activities in the specific location via a suite of biogeochemical reactions involving the N-containing molecules, plant root, soil, and microbes; Then, the glycosidase activity could increase or decrease in each location affected by N fertilizer input, leading to re-distribution of enzymes and other soil features (e.g., SOC and MBC), i.e., restructuring spatial heterogeneity in the plot level; As a consequence of spatial restructuring, the plot level mean or central tendency and variation were likely changed as well. The N fertilization-induced changes in spatial heterogeneity of soil enzymes will also likely depend upon the indigenous site condition and the legacy effect that it exerted for years to decades^75^.

In response to N fertilization, both central tendency and spatial heterogeneity of glycosidases contrasted among different enzymes. Besides the unique chemical characteristics of each enzyme, the indigenous variation of each enzyme in a plot may regulate the effect of N fertilization. Glycosidases showed medium CVs (i.e., 15–47%) as they appeared to be higher than that for soil moisture, total pore space, pH, SOC, TN, δ^13^C and δ^15^N^30,44,76^ and substantially lower than that for extractable soil Fe and Mn^52^. However, substantially different CVs were evident between enzymes in this study. For instance, *BG* showed the most extreme CVs of 15–47% while *AG* showed narrow CVs of 23–29% in GG. The contrasting plot-level variations led to a difference of sample size required up to an order of magnitude (123 vs. 14; Table 4). The same number of soil sampling design in each plot unavoidably induced differential error terms for different enzymes that could have contributed to the insignificant treatment effects for most enzymes. However, *BX* responded significantly to both N fertilization and crop type suggesting an essentially strong and a suite of high order interaction of this enzyme with plant and soil, mediated by microbial community strategy. Due to the complexity of interactions, the fertilization effects on spatial heterogeneity of *BG* and *CBH* were identified. This suggested that the N fertilization effects on central tendency and spatial distribution of enzymes decoupled for the same enzyme.

## Conclusions

Our study demonstrated that N fertilization significantly enhanced central tendency and resulted in more pronounced spatial heterogeneity of glycosidase activities in top soil horizons in bioenergy croplands, but the N fertilization effects varied with crop species and enzyme type. N fertilization significantly enhanced *BX* by 14–44% in SG, and consistently restructured the spatial heterogeneity of *BG* in SG and *CBH* in GG. Two bioenergy crops showed contrasting glycosidases’ activities (GG > SG) and their plot-level variations (GG < SG). The unique enzyme characteristics and their interactions with plant root and soil, mediated by soil microbial community, possibly explained the enzyme-specific responses under N fertilization. Though N fertilization elevated mean activities and spatial heterogeneity of glycosidases, these effects varied with crop species and enzyme type. Future studies should focus on specific enzyme when evaluating N fertilization effect in certain bioenergy cropland soil.

## Supplementary information

Supplementary Information.
