# Supplementary material for: Effects of nitrogen fertilization and bioenergy crop species on central tendency and spatial heterogeneity of soil glycosidase activities
Source: Sci Rep. 2020 Nov 12;10:19681. doi: 10.1038/s41598-020-76837-1 (PMC7664997; doi:10.1038/s41598-020-76837-1)
Supplement: Supplementary file 1 — Supplementary Information. [file 41598_2020_76837_MOESM1_ESM.docx]

**Supplementary Materials**

**Effects of** **Nitrogen Fertilization and** **Bioenergy** **Crop Species on** **Central Tendency and** **Spatial Heterogeneity of** **Soil Glycosidase Activities**

Min Yuan ^1,2,#^, Jianjun Duan^1,3,#^, Jianwei Li^1*^, Siyang Jian^1^, Lahiru Gamage^1^, Kudjo E Dzantor^1^, Dafeng Hui^4^, Philip A. Fay^5^

^1^ Department of Agricultural and Environmental Sciences, Tennessee State University, Nashville, TN 37209

^2^ Sichuan Provincial Academy of Natural Resource Sciences, Chengdu, 610015, Sichuan, China

^3^ Guizhou Provincial Key Laboratory for Tobacco Quality, College of Tobacco Science, Guizhou University, Guiyang 550025, Guizhou, China

^4^ Department of Biological Sciences, Tennessee State University, Nashville TN 37209

^5^ Grassland Soil and Water Research Laboratory, USDA ARS, Temple, TX 76502

**Table S1.** Significant regression coefficients of trend-surface analysis and coefficients of determination (r^2^) for *AG, BG, BX, CBH,* and *C_acq_* (µmol g^-1^soil h^-1^) under three N fertilization treatments (NN, LN and HN) in two bioenergy croplands (SG and GG). *, ** and *** represent significance at P < 0.05, 0.01 and 0.001, respectively. The abbreviations are referred to Table 1 and Table 2.
